# Drug safety in patients with mitochondrial disease: an observational cohort study

**DOI:** 10.1007/s10072-026-09298-5

**Published:** 2026-08-06

**Authors:** Oskar Järvinen, Mika H. Martikainen

**Affiliations:** 1 Clinical Neurosciences, Department of Clinical Medicine, University of Turku, Turku, Finland; 2 Neurocenter, Turku University Hospital, Turku, Finland; 3 Research Unit of Clinical Neuroscience, Neurology, University of Oulu, Oulu, Finland; 4 Department of Neurology, Neurocenter and Medical Research Center, Oulu University Hospital, OYS, P.O. Box 10, FIN-90029 Oulu, Finland

**Keywords:** Drug safety, Medication, Mitochondria, Mitochondrial disease, Pharmacological treatment

## Abstract

**Background:**

Mitochondrial diseases are common inherited neurometabolic disorders and frequently involve the nervous system, yet their multisystem nature often necessitates complex pharmacological management. Many commonly prescribed medications have off-target effects on mitochondrial function, and patients with mitochondrial disease may be particularly vulnerable to such effects due to impaired energy metabolism. However, systematic data on medication safety in this patient group remain scarce.

**Methods:**

In this retrospective, single-centre, cohort-based study at Turku University Hospital (Turku, Finland), we reviewed the medication data from all hospital stays and outpatient prescriptions of 44 mostly adult (20 women; mean age 50 years, range 12–83 years) patients with genetically and clinically confirmed mitochondrial disease for years 2010–2022. We used the Anatomical Therapeutic Chemical system for drug classification. Potential drug-drug interactions and potential adverse drug reactions were investigated. Special focus was on potential mitochondrial toxicity of drugs and clinically relevant drug–drug interactions.

**Results:**

Altogether ~ 1000 individual medication entries were reviewed. We identified several common drugs with potentially adverse effects on mitochondria, including metformin, beta-blockers, statins, ciprofloxacin, fluoxetine, ibuprofen, and certain anti-seizure drugs. Medications generally considered contraindicated in mitochondrial disease were not observed. No high-risk drug interactions were detected. Additional finding of clinical relevance was the frequent use of analgesics.

**Conclusions:**

Further research regarding mitochondrial safety of several drug classes is needed for more evidence-based safety evaluations. Pain in the context of mitochondrial disease merits increased attention.

**Supplementary Information:**

The online version contains supplementary material available at https://doi.org/10.1007/s10072-026-09298-5.

## Introduction

Mitochondria, the powerhouses of the cell, are intracellular organelles that produce most of the adenosine triphosphate (ATP) required by the cell and are irreplaceable for eukaryotic life. The main function of mitochondria is oxidative phosphorylation (OXPHOS) that takes place in the inner mitochondrial membrane and produces adenosine triphosphate (ATP) for cellular aerobic energy needs [1]. Other important functions of mitochondria include the control of cellular calcium levels, fatty acid oxidation, and regulation of apoptosis [2].

Mitochondria have their own mitochondrial DNA (mtDNA). Mutations in either nuclear or mitochondrial genomes can lead to mitochondrial dysfunction and cause mitochondrial disease [3]. With a prevelance of about 1 in 4300, mitochondrial diseases are the most common group of inherited metabolic disorders in humans [4]. Because mitochondria are essential organelles in all human cells, mitochondrial disease can affect all organs [5]. Myopathy, epilepsy, diabetes mellitus, sensorineural hearing loss, ataxia, and stroke-like episodes are among well-recognised features of mitochondrial disease [6].

Recognition of potentially toxic effects of drugs on mitochondria has increased in recent decades [7]. Pre-clinical models have shown that drug-induced mitotoxicity can be caused by inhibition of the electron transport chain complex, uncoupling of mitochondrial OXPHOS, disruption in the mtDNA replication and thus inhibition of mitochondrial protein synthesis, reactive oxygen species (ROS) formation and inhibition of mitochondrial fatty acid b-oxidation (FAO) [8, 9]. There are several drugs with well-known mitotoxic effects, and several drugs with suspected side effects caused by mitotoxicity, but where evidence is less clear.

Some recommendations and guidelines regarding drug safety in patients with mitochondrial disease have been presented previously [5, 8]. The use of aminoglycosides is recommended to be limited to emergency use only or by screening for mutations associated with both aminoglycoside-induced and non-syndromic hearing loss [10, 11]. Valproate is known to cause liver toxicity and steatosis through mitochondrial dysfunction [12]. It is contraindicated in patients with mitochondrial disease associated with *POLG* mutations or even a suspicion of POLG disease, because it can cause fatal liver failure [13, 14]. Also in other types of mitochondrial disease, valproate should only be used in exceptional situations. Neuromuscular blocking agents should be used carefully and under high supervision [5]. 

There are several further potential mitochondrial adverse effects of commonly used drugs. Statins may induce mitochondrial dysfunction, which may be a relevant mechanism underlying statin-associated myopathy [15]. Metformin, a drug commonly used to treat type 2 diabetes mellitus, has been linked to risk of mitochondrial dysfunction in in vitro studies [16]. However in practice, it is widely accepted and safe treatment also in patients with mitochondrial disease [8]. Metformin is secreted to urine through renal function and lactic acidosis is a potential adverse effect. Many patients with a mitochondrial disease have renal impairment and are also at higher risk of developing metabolic acidosis [8]. Tetracycline and ciprofloxacin antibiotics can inhibit mitochondrial function and lead to adverse effects [17]. Propofol-Related Infusion Syndrome (PRIS) is considered likely caused by the mitochondrial toxicity of propofol [18]. Patients with inhibited mitochondrial beta-oxidation are in danger of developing liver failure and the use of ibuprofen, amiodarone or tamoxifen can furthermore inhibit FAO [9]. Although experimental studies have suggested that L-dopa can induce oxidative stress and adversely affect mitochondrial respiratory chain function [19], these observations have not been confirmed in clinical settings. Moreover, the drug concentrations used in many in vitro experiments exceed those achieved with therapeutic dosing. Accordingly, L-dopa remains the standard and generally well-tolerated treatment for parkinsonism, including in patients with primary mitochondrial diseases [8]. The classical aromatic anti-seizure medications (e.g. phenobarbital and carbamazepine) can cause mitochondrial toxicity in vitro which can lead to idiosyncratic hepatic damage associated with this drug-class [20]. Several common antidepressants, including amitriptyline, escitalopram, fluoxetine, and sertraline, have negative effects on mitochondrial function in vitro [17, 21]. 

The spectrum and drug safety of medications used by individuals with mitochondrial disease is not well known. To evaluate the mitochondrial drug safety of such treatments, and to detect potential adverse effects or harmful drug-drug interactions, we reviewed the medications used in a well characterised, single-centre cohort of Finnish patients with mitochondrial disease.

## Materials and methods

In this retrospective, register-based study, we assessed the current and former medication history among a cohort of individuals previously diagnosed with mitochondrial disease at Turku University Hospital (TUH, Turku, Finland). There were no criteria for age or sex. Medication history data of each patient were obtained from TUH electronic patient records. Medication data from all hospital stays and outpatient prescriptions were collected for years 2010–2022. The current medication of each patient was obtained from the electronic patient records. Patients lost to follow-up with no information on recent medication were excluded from further analysis. For deceased patients, the latest available information of ther regular medication was used in the analysis.

### Data processing and analysis

We used the Anatomical Therapeutic Chemical (ATC) system to list and classify the drugs used by the patients. ATC is the most widely recognized classification system for drugs and recommended by the World Health Organization (WHO) [22]. In the ATC system, the active substances of the drugs are classified in a hierarchy with five different levels with 14 main (1st level) anatomical/pharmacological groups. The classification is based on the therapeutic and chemical characteristics of the substances and the organ or system on which they act [23]. However, although many drugs are used for more than one indication, usually only one ATC code is assigned. In the analysed patient data, we labeled every individual drug entry with a corresponding ATC code. The drugs with no ATC code (e.g. *ex tempore* prescriptions) were excluded from further analysis.

Potential drug-drug interactions (pDDIs) and potential adverse drug reactions (ADRs) were investigated using an online database Riskbase/Inxbase (Medbase Ltd., Turku, Finland) [24], commonly used by medical professionals in Finland and also utilised for research purposes [25–27]. Riskbase describes medication side effects for over 1800 drugs in 11 different categories. Inxbase describes over 35,000 different harmful drug interactions with classification for scientific evidence and clinical relevance. Inxbase divides pDDIs into four categories based on the potential risk to health: D—high risk, combination should be avoided; C—moderate risk, combination can be used but dose adjustment might be needed; B—unclear risk; A—clinically insignificant risk. Riskbase divides the potential ADRs into ten groups: bleeding risk, constipation, anticholinergic effect, orthostatism, prolonging of the QT interval, renal toxicity, sedation, seizure risk, serotonergic effect, and potassium level. Riskbase then calculates a risk score in all 10 groups as follows: D—high risk; C—moderate risk; B—slightly increased risk; A—no known risk. The high-risk category on Riskbase does not contraindicate using the medicine but defines the probability of risk. Analysis based on Riskbase/Inxbase was applied retrospectively to the treatment regimens of all patients in the study.

## Results

We identified 44 patients (24 men, 20 women) with mitochondrial disease for whom medication data were available. The current age of the subjects ranged between 12 and 83 years (average 50 years, median 55 years). Nine of the patients (five men, four women) were deceased at the time of the study. There were altogether ~ 1000 individual medication entries. The genetic causes of mitochondrial disease are outlined in Table 1.

**Table 1:** The genetic causes of mitochondrial disease in the studied patients

Genetic variant	*N*
mtDNA variants	
m.3243 A > G	18
m.8344 A > G	5
mtDNA deletion	3
Other mtDNA variants	5
nDNA variants	
*POLG* variants	4
Other nDNA variants	9
Total	44

*mtDNA* mitochondrial DNA, *nDNA* nuclear DNA

We recorded all medications noted in patient data and sorted these according to the ATC classification (Table 2). Based on previous literature, we detected among the patients’ medications the following drugs: metformin in five patients (11%), beta blockers in 11 patients (25%), statins in eight patients (18%), ciprofloxacin (black box warning) in three patients (7%), ibuprofein in 24 patients (55%), fluoxetine (black box warning) in one patient (2%). Altogether 14 patients (32%) used some antiseizure medication (ASM). Some central neuromuscular blocking agents were used by seven patients (16%). These drugs have been suggested to have potentially negative effects on mitochondrial function based on preclinical data or theoretical considerations, which is however not evidence that they would be associated with clinically relevant mitochondrial toxicity or unsafe to use. No patient used aminoglycosides, periferial neuromuscular blocking agents, or valproate, with well known harmful effects on mitochondria [5]. We also looked in detail at medications that primarily affect the central nervous system (CNS) as well as medications used to treat diabetes mellitus and hypercholesterolaemia (Table 3).

**Table 2:** Used medications according to the ATC classification

ATC	Category	Patients, *N* (%)
A	Alimentary tract and metabolism^1^	37 (84)
B	Blood and blood forming organs	21 (48)
C	Cardiovascular system^2^	20 (45)
D	Dermatologicals	3 (7)
G	Genito urinary system and sex hormones	11 (25)
H	Systemic hormonal preparations, excluding sex hormones and insulins^3^	12 (27)
J	Anti-infective for systemic use	28 (64)
L	Antineoplastic and immunomodulating agents	3 (79
M	Musculo-skeletal system	28 (64)
N	Nervous system^4^	39 (89)
P	Antiparasitic products, insecticides, and repellents	10 (23)
R	Respiratory system	21 (48)
S	Sensory organs	16 (36)
V	Various	2 (7)

*ATC* anatomical therapeutic chemical

^1^Most commonly drugs for acid-related disorders and drugs used in diabetes mellitus (*N* = 18; insulin *N* = 17)

^2^Most commonly beta blocking agents, agents acting on the renin–angiotensin system, and lipid lowering medication

^3^Most commonly systemic corticosteroids

^4^Most commonly paracetamol (*N* = 30), strong opioids (*N* = 21), and levetiracetam (*N* = 10)

**Table 3:** Potentially mitotoxic medications used

ATC codes		Drug/drug class	Patients (*N*)	
Table A. Nervous system				
N01A		Alfentanil	1	
N01B		Lidocaine	10	
N02A		Oxycodone	21	
		Oxycodone + naloxone	3	
		Fentanyl	1	
		Codeine + paracetamol	8	
		Tramadol	4	
N02B		Paracetamol	30	
		Pregabalin	4	
		Gabapentin	5	
N02CA		Ergostamine-Belladonna- Phenobarbital	1	
N02CC		Triptans	3	
N03		Phenobarbital	1	
		Phenytoin	1	
		Fosphenytoin	1	
		Clonazepam	8	
		Carbamazepine	1	
		Oxcarbamazepine	2	
		Vigabatrin	1	
		Lamotrigine	3	
		Topiramate	4	
		Levetiracetam	10	
		Lacosamide	4	
N04		Pramipexole	1	
N05A		Antipsychotics	12	
N05BA		Benzodiatzepine derivatives	22	
		Diazepam	14	
		Oxazepam	6	
		Lorazepam	8	
		Clobazam	2	
		Aprazolam	1	
N05BB		Diphenylmethane derivatives	3	
		Hydroxyzine	3	
N05C		Hypnotics and sedatives	19	
		Temazepam	6	
		Midazolam	5	
		Zopiclone	6	
		Zolpidem	2	
N06A		Antidepressants	10	
N06B		Psychostimulants	4	
Table B. Drugs used in diabetes				
A10A	Human Insulin		7	
	Insulin Aspart		12	
	Insulin Glulisine		1	
	Insulin Lispro		2	
	Insulin Glargine		10	
	Insulin Detemir		4	
	Insulin Degludec		4	
A10B	Metformin		5	
	Glimepiride		1	
	Sitagliptin		4	
	Linagliptin		3	
	Dapagliflozin		4	
Table C. Lipid lowering agents				
C10A	Simvastatin		4	
	Atorvastatin		5	
	Rosuvastatin		2	
	Fenofibrate		1	
	Ezetimibe		5	

*ATC* anatomical therapeutic chemical

We observed 13 clinically significant class C and D drug-drug interactions in 5 patients. Potential harmful drug side effects found in the patients’ current medications were as follows: anticholinergic in four patients (9%), bleeding risk in four (9%), potassium imbalance in nine (20%), sodium imbalance in 11 (25%), orthostatism in six (14%), lengthened QT time in one (2%), sedation in 10 (23%), serotonergic in two (5%), and constipation in eight cases (18%). No medication induced risk of seizures or nephrotoxicity were found. This kind of drug interactions are also common in the general population, and these data do not depict drug-drug interactions particularly affecting patients with mitochondrial disease (Supplementary table S1).

## Discussion

In this single-centre study, we reviewed the complete medication data of 44 patients with mitochondrial disease. From altogether around 1000 individual medication entries, we identified individual drugs with potential or theoretical negative effects on mitochondria as well as more widely used drug classes whose adverse effect profile may be more complex in patients with mitochondrial disease. We found no use of valproate or aminoglycosides, with potentially very severe adverse effects in mitochondrial disease. However, for several drugs, the potential mitochondrial adverse effects or mitochondrial toxicity are unknown and available information is based on in vitro and in vivo pre-clinical studies and case reports. The results of pre-clinical studies where mitotoxicity is typically investigated in healthy cell lines are difficult to extrapolate to patients affected by mitochondrial dysfunction. This highlights the need for studies that specifically focus on potential negative drug-related events in patients with mitochondrial disease [8]. 

In 13 (33%) patients some pDDI was observed and for 5 (13%) there was a class C or D interaction with another drug. For comparison, in a study conducted with first-time hospitalized myastenia gravis (MG) patients [28], some kind of drug-drug interaction was detected in 89% of patients. In our study, the most common class C interactions were commonly used drugs and supplements e.g., calsium, magnesium and proton-pump-inhibitors decreasing absorption of bisphosphonate and thyroxin. The only class D interaction was with ergostamine, ebastine, and phenobarbital which was used as an ASM. Overall, we found few serious pDDIs in this cohort. The drug interaction and adverse effect profiles of commonly used medications in patients with mitochondrial disease are similar to those in the general population, and almost all medications can be prescribed according to standard clinical practice, as also suggested by recent consensus statement [8]. However, as the use of several medications is common in patients with mitochondrial disease, meticulous attention for possible DDIs is needed.

Sodium and potassium imbalance, sedation, and constipation were the most common potential side effects. These are also prevalent side effects in the general population, partly because of e.g. common use of antihypertensive medications and opioids. We did not observe nephrotoxicity or increased seizure risk related to medications. In one patient, the current medication, which included escitalopram and amitriptyline had a side effect of possibly prolonging QT time.

Statin use in our study (18%) was comparable to previous studies involving patients with mitochondrial disease (14%) [29]. Patients with mitochondrial dysfunction may be more vulnerable to statin induced myopathy [30]. Antidepressant use was also common (23%). Previous studies highlighted possibility of some antidepressants negative effect on mitochondria [17], but data on antidepressant use in patients with mitochondrial disease is scarce. Antibiotic selection in this patient group has been little studied, despite reports of negative effects of certain antibiotics on mitochondrial function [31, 32]. In our study, three patients had used ciprofloxacin, but there were no data suggesting use of tetracyclins.

In our study, 39% of the patients used insulin and 11% used metformin. Despite some in vitro studies and theoretical arguments suggesting that metformin could have a negative effect on mitochondrial function, its use is likely to be safe in patients with mitochondrial disease and diabetes. Indeed, anecdotal evidence suggests it is useful and well tolerated in particularly overweight patients [33]. In our study, we were not able to trace the individual decisions that guided the use of metformin in these patients. In mitochondrial disease, the overall prevalence of epilepsy is reported to be 10–23%, with 20–50% of patients experiencing seizures over the course of the disease. Our founding of 32% ASM usage rate was in line with previous findings [34, 35]. We observed some use of ASMs with potentially adverse effect on mitochondria (e.g., carbamazepine, phenobarbital), although the use of these medications is to some extent accepted among experts in this field [8]. Among the patients in this study, 50% had used benzodiazepine medication at some point. There are diverse indications for benzodiazepine use, however at least for oxazepine and alprazolam it is arguably unlikely that they have been used as ASMs. The extent and causes of benzodiazepine use in patient with mitochondrial disease should be investigated further because of the well known potential adverse effects related to their long term use [36, 37].

A considerable part (68%) of the patients used some pain medication. 55% of the patients had at some point used an opioid medication. In contrast, the usage rate of several drugs commonly used to treat neuropathic pain (including gabapentinoids, duloxetine, venlafaxine, amitriptyline, and nortriptyline) was only 16%. Previous study found a high prevalence (67%) of chronic, mostly neuropathic, pain in patients with mitochondrial disease [38]. These previous data and our results suggest that pain is not uncommon in patients with mitochondrial disease, and this phenomenon probably warrants more attention.

In conclusion, we did not observe use of clearly harmful medications or severe DDIs among these patients with mitochondrial disease. Despite some evidence of potentially or theoretically harmful mitochondrial effects in several medications, there are few data to suggest that these would be reflected in some relevant clinical adverse effects in patients with mitochondrial disease.

## Supplementary Information

Below is the link to the electronic supplementary material.

Supplementary Material 1 (PDF 40.2 KB)

## Data Availability

The individual patient data used in this study are not publicly available. Aggregate data are available from the corresponding author upon reasonable request.
