# Supplementary material for: Drug safety in patients with mitochondrial disease: an observational cohort study
Source: Neurol Sci. 2026 Aug 6;47(9):683. doi: 10.1007/s10072-026-09298-5 (PMC13447329; doi:10.1007/s10072-026-09298-5)
Supplement: Supplementary file 1 [file 10072_2026_9298_MOESM1_ESM.pdf]

Supplementary Table S1. Potentially significant drug interactions.

Interaction	Drug 1	Drug 2	Mechanism	N
C	Calcium	Bisphosphonate	Decreased absorption of bisphosphonate	2
C	Calcium	Levothyroxine	Decreased absorption of levothyroxine	2
C	Pantoprazol	Levothyroxine	Decreased absorption of levothyroxine	2
C	Magnesium	Levothyroxine	Decreased absorption of levothyroxine	1
C	Simvastatin	Warfarin	Increased anticoagulation effect	1
D	Ergostamine	Phenobarbital	Ergostamine concentration decreases, treatment possibly fails	1
D	Ebastine	Phenobarbital	Ebastine concentration decreases, treatment possibly fails	1
C	Vitamine D	Phenobarbital	Decreased concentrations of vitamin D	1
C	Phenobarbital	Simvastatin	Decreased therapeutic effect in simvastatin	1
C	Azathioprine	Sulfasalazine	Increased thiopyridinetoxicity and myelosuppression risk	1

Interactions are classified according to the Inxbase/Riskbase classification[1]. The first column illustrates the interaction probability. D—high risk, combination should be avoided; C—moderate risk, combination can be used but dose adjustment might be needed.

[1]Böttiger Y, et al. SFINX-a drug-drug interaction database designed for clinical decision support systems. Eur J Clin Pharmacol. 2009 Jun;65(6):627-33. doi: 10.1007/s00228-008-0612-5. PMID: 19205683.
